# Enhancement of antibiotics antimicrobial activity due to the silver nanoparticles impact on the cell membrane

**DOI:** 10.1371/journal.pone.0224904

**Published:** 2019-11-08

**Authors:** R. Vazquez-Muñoz, A. Meza-Villezcas, P. G. J. Fournier, E. Soria-Castro, K. Juarez-Moreno, A. L. Gallego-Hernández, N. Bogdanchikova, R. Vazquez-Duhalt, A. Huerta-Saquero

**Affiliations:** 1 Centro de Nanociencias y Nanotecnología, Universidad Nacional Autónoma de México, Ensenada, Baja California, México; 2 Centro de Investigación Científica y de Educación Superior de Ensenada, Ensenada, Baja California, México; 3 Instituto Nacional de Cardiología Ignacio Chávez, Ciudad de México, México; 4 Departamento de Investigación en Física, Universidad de Sonora, Hermosillo, Sonora, México; Institute of Materials Science, GERMANY

## Abstract

The ability of microorganisms to generate resistance outcompetes with the generation of new and efficient antibiotics; therefore, it is critical to develop novel antibiotic agents and treatments to control bacterial infections. An alternative to this worldwide problem is the use of nanomaterials with antimicrobial properties. Silver nanoparticles (AgNPs) have been extensively studied due to their antimicrobial effect in different organisms. In this work, the synergistic antimicrobial effect of AgNPs and conventional antibiotics was assessed in Gram-positive and Gram-negative bacteria. AgNPs minimal inhibitory concentration was 10–12 μg mL^-1^ in all bacterial strains tested, regardless of their different susceptibility against antibiotics. Interestingly, a synergistic antimicrobial effect was observed when combining AgNPs and kanamycin according to the fractional inhibitory concentration index, FICI: <0.5), an additive effect by combining AgNPs and chloramphenicol (FICI: 0.5 to 1), whereas no effect was found with AgNPs and β-lactam antibiotics combinations. Flow cytometry and TEM analysis showed that sublethal concentrations of AgNPs (6–7 μg mL^-1^) altered the bacterial membrane potential and caused ultrastructural damage, increasing the cell membrane permeability. No chemical interactions between AgNPs and antibiotics were detected. We propose an experimental supported mechanism of action by which combinatorial effect of antimicrobials drives synergy depending on their specific target, facilitated by membrane alterations generated by AgNPs. Our results provide a deeper understanding about the synergistic mechanism of AgNPs and antibiotics, aiming to combat antimicrobial infections efficiently, especially those by multi-drug resistant microorganisms, in order to mitigate the current crisis due to antibiotic resistance.

## Introduction

Infectious diseases caused by pathogenic bacteria are one of the most common causes of death worldwide and a constant health risk in all countries [1]. Today, the burden of infectious disease on health, economy and other social aspects is so complex, that the worldwide cost cannot be estimated [2]. Current antimicrobial therapies have significant disadvantages, such as their limited diversity, antagonistic interaction, and the effects of unfinished antibiotic treatments leading to the acquisition of resistance of microorganisms, among many others [3].

Multi-resistant bacteria constitute one of the most severe worldwide public health problems [4], and new resistant strains frequently appear decreasing the efficacy of current treatments leading to severe public health risks [5,6]. It also negatively impacts several fields of human activity, such as agriculture, aquaculture, and veterinary [5,7]. Unfortunately, the evolution of multi-resistant bacteria has overcome the development rate of new antibiotics; therefore, the generation of new and efficient antimicrobial treatments are critical [8].

Strategies to preserve antibiotic effectiveness have been recommended [5]. One of the alternatives to fight against multi-drug resistant organisms is the use of nanoantibiotics (nanomaterials with antimicrobial properties [9,10]). Due to their antiviral and antibacterial properties, silver nanoparticles (AgNPs) are the most promising nanoantibiotics nowadays [11–15]. The advantages of AgNPs are a generalized mode of action against different pathogens, such as viruses, bacteria and fungi; as well as their antibacterial effectiveness regardless the microbial susceptibility to conventional antibiotics, including efflux pumps and biofilm formation [16,17], thus AgNPs can overcome the microorganisms resistance to conventional antibiotics [18]. Moreover, the use of AgNPs has different advantages beyond their antiviral and antimicrobial activity. AgNPs can be synthesized by facile, non-expensive and environmental friendly methods, and they can be produced using chemical processes or by biosynthesis [19–21]. The AgNPs showed a multi-level mode of action on bacterial cells affecting metabolic processes: A) cell wall and membrane disruption and cell permeability increase [22–25]; B) AgNPs penetration and intracellular damage disrupting metabolic pathways [24,26,27]; C) biomolecules damage (DNA, proteins) [25]; and D) reactive oxygen species generation [28–30]. For recent reviews see [31,32].

Recently, an improvement in antimicrobial activity–synergistic effect- has been reported when AgNPs are combined with several antibiotics, such as ampicillin, amoxicillin, and chloramphenicol [33–38]. On the contrary, reports have shown an antagonistic interaction between AgNPs and amoxicillin or oxacillin [37]. Studies focused on the mechanism of action of the NPs-antibiotic combinations have suggested that the improvement in the antimicrobial activity could be due to their chemical interaction, however, the underlying molecular mechanism of the effect, either synergistic or antagonistic, still requires clarification [34,36,39].

Given the presence of contradictory reports, it was of our interest to evaluate the antimicrobial activity of AgNPs combined with antibiotics of different cellular targets and therefore, different mechanisms of action. We selected chloramphenicol (Cm), kanamycin (Km), ampicillin (Amp), aztreonam (Azm) and biapenem (Bpm). The enhanced effectiveness of AgNPs-antibiotics combinations was characterized by different analytical techniques in order to elucidate the antimicrobial mechanisms involved. Since synergy is highly relevant for antimicrobial combinatorial therapies, we explored both Gram-positive and Gram-negative bacteria. We were able to determine that AgNPs alter the cell membrane integrity and potential, which increase cell permeability and facilitate antibiotics intracellular access, increasing the efficiency of those antibiotics with intracellular targets.

## Material and methods

### Characterization of silver nanoparticles (AgNPs)

Argovit AgNPs were obtained from Vector Vita Ltd (Novosibirsk, Russia). They were stabilized using polyvinylpyrrolidone (PVP). AgNPs were characterized by physicochemical methods (S1 Fig). Briefly, the surface plasmon resonance was characterized by UV-Vis spectrophotometry (Multiskan Go, Thermo Scientific) in a range from 270 to 600 nm. The morphology and chemical composition of AgNPs were examined in a TEM (Jeol JEM 2100). Also, elemental composition analysis (EDX) was performed to confirm the presence of silver. Their average hydrodynamic diameter and stability of AgNPs were measured by Dynamic Light Scattering (DLS) using a Zeta sizer nano [40].

### Bacterial strains and culture conditions

Experiments were performed on four bacterial strains. Gram-negative: *Escherichia coli* DH5α (non-pathogenic) and *Salmonella enterica* serovar Typhimurium ATCC SC14028 (*S*. Typhimurium) (pathogenic); Gram-positive: *Staphylococcus aureus* (clinical isolate, pathogenic) and *Bacillus subtilis* (non-pathogenic), from the collection kept at the Centro de Nanociencias y Nanotecnologia—Universidad Nacional Autónoma de México. For experiments, all bacteria were grown in Muller-Hinton (MH) broth. For MH plates, 1.5% of agar was added. Cultures were incubated for 24 h, 37°C and 180 rpm.

### Antimicrobial agents

Antibiotics: chloramphenicol (Cm), kanamycin (Km), biapenem (Bpm), and aztreonam (Azm) were obtained from Sigma-Aldrich (USA), and ampicillin (Amp) from Duchefa Biochemie (USA). The concentration range for antibiotics was selected based on the CLSI M100 protocol guidelines [41] and Vazquez-Muñoz, *et*. *al*., 2017 for AgNPs [42]. Minimal Inhibitory Concentrations (MIC) was determined for AgNPs and all the antibiotics used in this study (Table 1). Sublethal concentrations for combinatorial effect assays were selected based on MIC (S1 Table).

**Table 1:** Minimal inhibitory concentrations (MIC) for each organism. Concentration expressed in μg.mL^-1^.

Organisms	AgNPs	AgNO_3_	Cm	Km	Amp	Bpm	Azm
*E*. *coli*	10	6	0.75	0.1	2	0.05	1
*S*. Typhimurium	10	7	1	0.25	0.5	0.05	0.05
*S*. *aureus*	12	7	0.1	32	0.5	0.05	0.05
*B*. *subtilis*	11	7	16	0.25	0.1	0.01	32

Cm = chloramphenicol; Km = kanamycin; Amp = ampicillin; Bpm = biapenem, Azm = aztreonam. Results are the mean of at least three different experiments. Standard deviations did not exceed 5%.

### Antimicrobial activity of AgNPs, AgNO_3_ and antibiotics

The minimal inhibitory concentration (MIC) and sublethal concentrations were determined for AgNPs, AgNO_3_, and antibiotics. MIC was assessed according to the M07-A9 protocol of the Clinical Laboratory Standards Institute [43], with some modifications. Sublethal concentrations were optically determined by UV-Vis spectrophotometry, at a wavelength of 600 nm. As control, for each AgNPs and AgNO_3_ concentration tested, a solution without inoculum was used.

### Combined AgNPs-antibiotic treatments

The combined activity of sublethal concentrations of AgNPs (6 μg.mL^-1^) with different antibiotics was evaluated using the microdilution method. For each antibiotic, the sub-lethal concentrations used were half the MIC (S1 Table). As control, bacteria were exposed separately to the sub-lethal concentration of AgNPs and antibiotics respectively. Cells were incubated under standard laboratory conditions (37°C and 180 rpm), in 96-multiwell plates for 24 hrs. Bacterial growth inhibition was determined spectrophotometrically at a wavelength of 600 nm. To determine the interactive effect of antibiotics and AgNPs, the fractional inhibitory concentration index (FICI) modify by Odds [44], was used. FICI considers that paired combinations of agents can exert inhibitory effects that are more than the sum of their effects alone (synergy; FICI ≤0.5), or to less than the sum of their effects alone (antagonism; FICI >4) [44]. For this study, an additive effect was considered when FICI value is >0.5 and ≤1. FICI was determined by calculating the percentages of bacterial growth inhibition of AgNPs and antibiotics using the formula:

$$FICI=\frac{AgNPs\;growth\;inhibition\;alone+antibiotic\;growth\;inhibition\;alone}{growth\;inhibition\;of\;combined\;agents}$$

### AgNPs-antibiotic interaction characterization by optical analysis

Combined AgNPs-antibiotics treatments were analyzed by Fourier Transform Infrared (FT-IR) spectroscopy to assess a potential chemical interaction between AgNPs and antibiotics. Combined treatments were incubated in deionized water under standard conditions and then lyophilized. Finally, the samples were centrifuged and pellet mixed with potassium bromide (KBr). FT-IR analysis was performed on a Nicolet 6700, Thermo Scientific^®^ FT-IR spectrometer, in a range from 4000 to 400 cm^-1^.

Dynamic Light Scattering (DLS) analysis was performed to evaluate the stability of AgNPs when exposed to antibiotics based on changes in aggregation (hydrodynamic diameter) and charge (Z potential). AgNPs (50 μg.mL^-1^) mixed with antibiotics (100 μg.mL^-1^) were diluted in deionized water and measured at 0 h and 24 h, in culture conditions (37°C, 180 rpm). DLS analysis was performed in a Zetasizer Nano ZS system (Malvern).

### Ultrastructural analysis by Transmission Electron Microscopy (TEM)

The effect of AgNPs on bacterial cell wall structure and their probable bioaccumulation were evaluated by electron microscopy. *E*. *coli*, *S*. *aureus*, *S*. Typhimurium and *B*. *subtilis* were exposed to sub-lethal concentrations of AgNPs and AgNO_3_ (6 and 4 μg.mL^-1^, respectively), and incubated under standard conditions. Subsequently, bacterial cells were centrifuged, fixed with glutaraldehyde (2%), and post-fixed with OsO_4_ (1%). Samples were dehydrated and infiltrated in Spurr´s resin. Polymerized samples were sectioned (100 nm) in an ultramicrotome PowerTome X (RMC Boeckeler). Slides were mounted in 300 mesh copper grids covered with lacey carbon (Ted Pella). Ultrastructural analysis was performed with a TEM Jeol JEM-2010 (CNyN, UNAM) and a TEM Jeol JEM-1230 at 80 KeV (Pathology department, Cardiology National Institute). On the other hand, the AgNPs-antibiotic interaction was evaluated by mixing AgNPs and antibiotics separately for 24 hrs. AgNPs (50 μg.mL^-1^) mixed with antibiotics (100 μg.mL^-1^) were diluted in deionized water. One drop of the mixture was placed in 300 mesh copper grids covered with lacey carbon (Ted Pella), dried and observed by TEM.

### Effect of AgNPs on bacterial cell membrane potential and permeability

The effect of AgNPs on cell membrane polarity was evaluated according to the protocol suggested by Novo and Perlmutter [45]. Briefly, each bacterial strain (*E*. *coli*, *S*. Typhimurium, *S*. *aureus* and *B*. *subtilis*) were cultured under standard conditions and adjusted to 1 x 10^6^ cells per mL in sterile 1x PBS. Bacterial cultures were exposed to 10 μg.mL^-1^ of AgNPs or AgNO_3_ and stained with 18 μM DiOC6 (Sigma-Aldrich, USA). Positive control samples were exposed to 50 μM CCCP (Sigma-Aldrich, USA). Cells were incubated at room temperature for 1 h and analyzed in an Attune Flow Cytometer (Thermo Scientific), with a laser emitting at λ = 488 nm. Fluorescence was collected using the green and red channels. The forward scatter, side scatter, and fluorescence data were collected with logarithmic signal amplification. To evaluate membrane permeability, each strain was cultured under same conditions and exposed to AgNPs or AgNO_3_ as described above. The cells were stained with alamarBlue cell viability reagent (Thermo Scientific) and 75 nM of propidium iodide. Cells were incubated at 37°C for 1 h and analyzed by flow cytometry with a violet and blue laser emitting at λ = 405 and 488 nm, respectively. Fluorescence emission was recorded with the VL3 and BL3 channels as described before.

## Results

### Characterization of Silver nanoparticles (AgNPs)

AgNPs of Argovit formulation used in this study contains 1.2% of metallic silver stabilized with 18.8% of polyvinylpyrrolidone (PVP) in water (Vector-Vita Ltd). UV-Vis profile of AgNPs revealed a peak at 400 nm, which corresponds to the typical surface plasmon resonance of AgNPs. TEM micrographs showed that AgNPs were spheroid in shape, and their average diameter was 35 ± 15 nm. Elemental composition analysis (EDX) confirmed the presence of silver. Their average hydrodynamic diameter was 70.4 ± 0.5 nm, and their zeta potential value was -18 ± 1.1 mV. (S1 Fig). AgNPs concentration was calculated according to the metallic silver content.

### Minimal Inhibitory Concentrations (MIC) of AgNPs, AgNO_3_ and antibiotics

Both Gram-negative and Gram-positive bacterial strains were incubated with different antimicrobial concentrations to obtain minimal inhibitory concentration (MIC) and sublethal concentrations. *E*. *coli*, *S*. Typhimurium, *B*. *subtilis* and *S*. *aureus* were inhibited at concentration range from 10 to 12 μg.mL^-1^ of AgNPs and 6–7 μg.mL^-1^ of AgNO_3_. In the contrary, all strains showed notably different susceptibility to the antibiotics tested, with MICs ranging from 0.05 to 32 μg.mL^-1^ (Table 1). According to the Clinical and Laboratory Standars Institute (CLSI) criteria in the M100-S17 guidelines [46], our results showed *S*. *aureus* resistance only to Km (32 μg.mL^-1^), while *B*. *subtilis* was resistant to Cm and Azm (16 and 32 μg.mL^-1^, respectively). *E*. *coli* and *S*. Typhimurium susceptibility was detected to all antibiotics assayed (<2 μg.mL^-1^). Interestingly, MBC and MIC results were the same for all the bacterial strains in AgNPs and AgNO_3_ treatments (data not shown).

### Antimicrobial activity of combined AgNPs-antibiotic treatments

Once MIC values were determined for the all antimicrobial agents, sub-lethal concentrations of the combined AgNPs-antibiotics treatments were evaluated in order to identify a synergistic effect. The sublethal concentration used for AgNPs was 6 μg.mL^-1^, and half the MIC for each antibiotic (S1 Table). As control, bacteria were exposed separately to the sub-lethal concentration of AgNPs and antibiotics.

**Fig 1:**
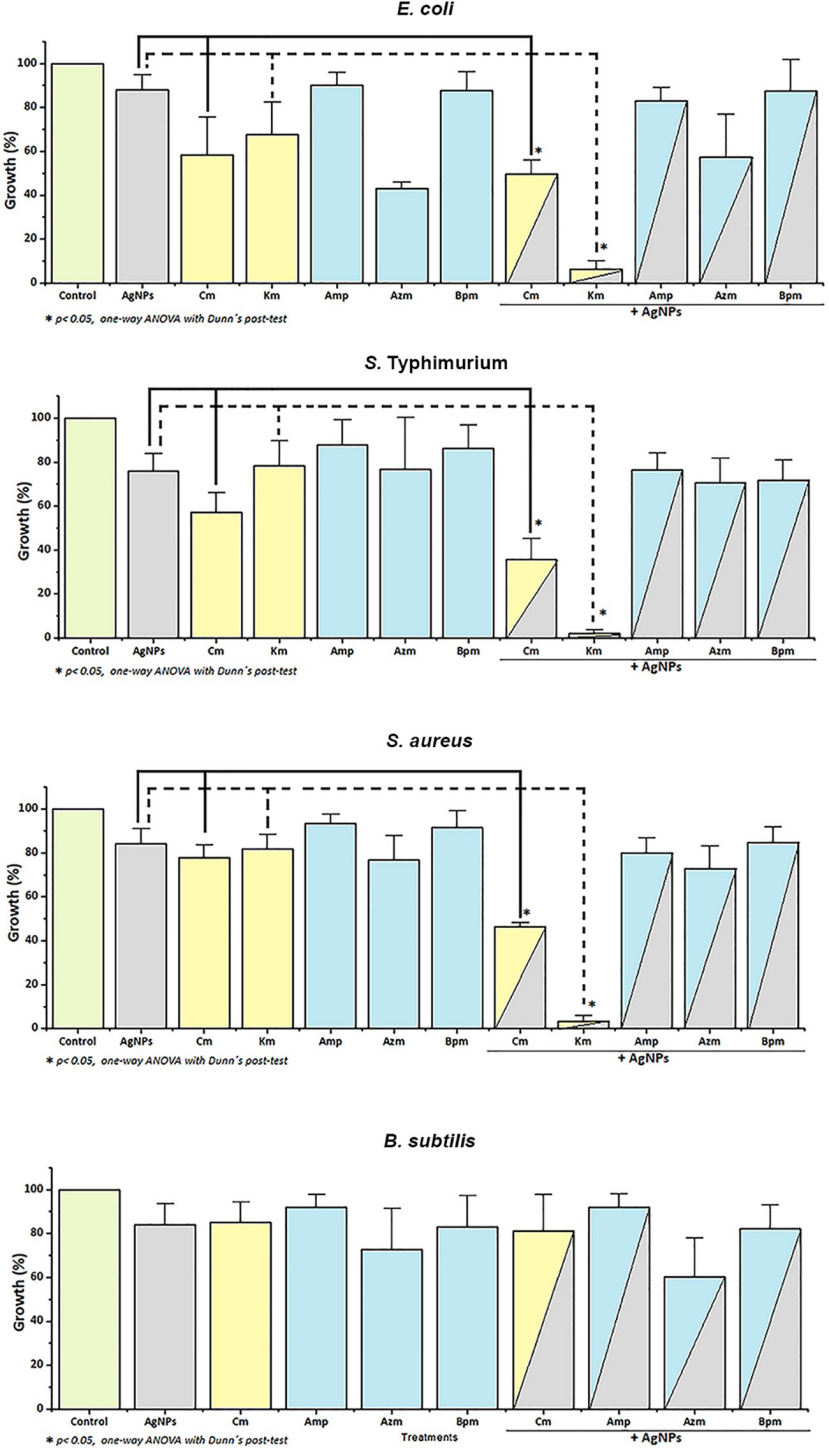
Effect on antimicrobial activity of the combined AgNPs-antibiotic treatments of *E*. *coli*, *S*. Typhimurium, *S*. *aureus*, and *B*. *subtilis*. Experiments were performed in triplicates. Significant changes are marked with asterisk: *p-value: <0.05.

The AgNPs-Cm combination caused around 50% growth inhibition on *E*. *coli*, *S*. Typhimurium, and *S*. *aureus*, whereas AgNPs-Km caused a dramatic growth inhibition on the same strains (around 95%) (Fig 1). The fractional inhibitory concentration index (FICI) was calculated for each AgNPs-antibiotic combination [44]. For this study, synergy is reported in cases were FICI ≤0.5, an additive effect when FICI value is between 0.5 and 1, and antagonistic with FICI >4. No effect was considered for FICI >1 to <4. Interestingly, statistical differences were observed when combining AgNPs with either Km or Cm in *E*. *coli*, *S*. Typhimurium and *S*. *aureus*, respectively (Fig 1 and Table 2). However, a synergistic effect was only observed for Km combinations while only an additive effect was identified for AgNPs-Cm. No effect was observed when combining neither AgNPs with β-lactam antibiotics for any microorganism tested, nor any treatment in *B*. *subtilis* (Fig 1).

**Table 2:** Fractional inhibitory concentration index (FICI) of combined antibiotics and AgNPs treatments.

Organisms	Combined treatment	FICI	Effect
*E*. *coli*	AgNPs + Cm	1.06	Additive
	AgNPs + Km	0.46	Synergistic
*S*. Typhimurium	AgNPs + Cm	1.05	Additive
	AgNPs + Km	0.47	Synergistic
*S*. *aureus*	AgNPs + Cm	0.69	Additive
	AgNPs + Km	0.35	Synergistic

Synergy ≤0.5; additive 0.5 to 1; no effect >1 to 4 and antagonistic >4. Cm = chloramphenicol. Km = kanamycin.

### DLS and FT-IR spectroscopic characterization of the combined AgNPs and antibiotic treatments

To determine whether there is a direct interaction between AgNPs and antibiotics, we performed an FT-IR and DLS analysis of the different antibiotics and the AgNPs, either in single or combined treatments. The FT-IR transmittance profiles of the combined AgNPs-antibiotic treatments had the characteristic peaks of both the PVP-AgNPs and the standard antibiotic (S2 Fig). Thus, the transmittance spectra analysis showed no evidence of a covalent chemical bonding between AgNPs and antibiotics.

On the other hand, DLS analysis showed that Km and Amp exert an effect on AgNPs size and charge (S3 Fig). Changes in size and charge may be due to electrostatic interactions between AgNPs and antibiotics, where Km and Amp could be absorbed around the AgNPs or inducing their agglomeration. In some cases, these differences were evident with a color change in the AgNPs-antibiotics mixture (data not shown). In order to clarify the DLS results, we analyzed the size and agglomeration state of the AgNPs when combining with Km and Amp by Transmission Electron Microscopy (TEM). In agreement with DLS data, AgNPs agglomeration was observed for AgNPs-Km and AgNPs-Amp mixtures, whereas no agglomeration was observed when combining AgNPs with Cm or Azm (S4 Fig).

### Effect of AgNPs on cell ultrastructure

**Fig 2:**
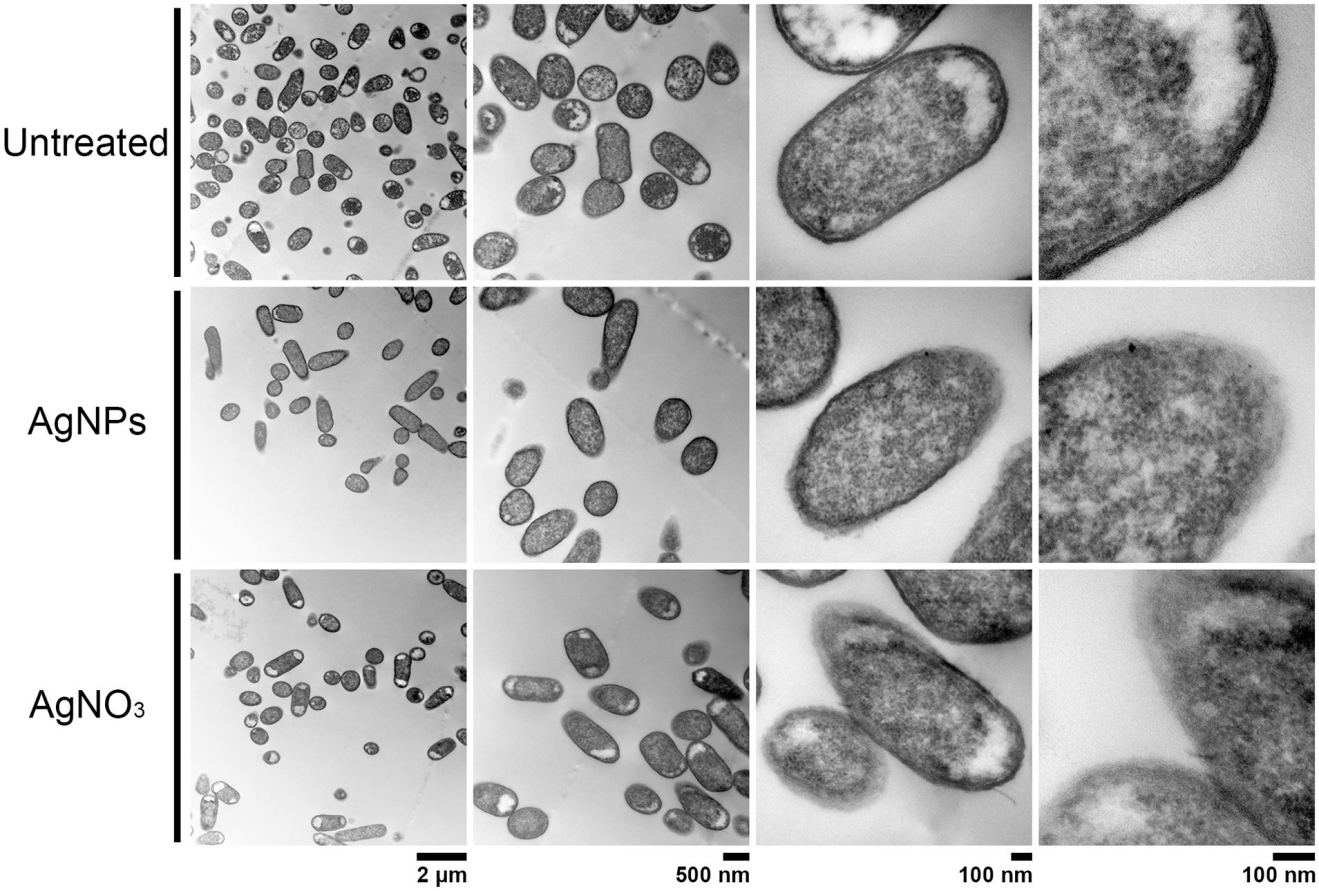
Structural analysis by Transmission Electron Microscopy (TEM) of *E*. *coli* cells exposed to AgNPs or AgNO_3_ (6 and 4 μg.mL^-1^, respectively). Untreated cells were used as control. Representative images of 3 biological replicates are shown.

**Fig 3:**
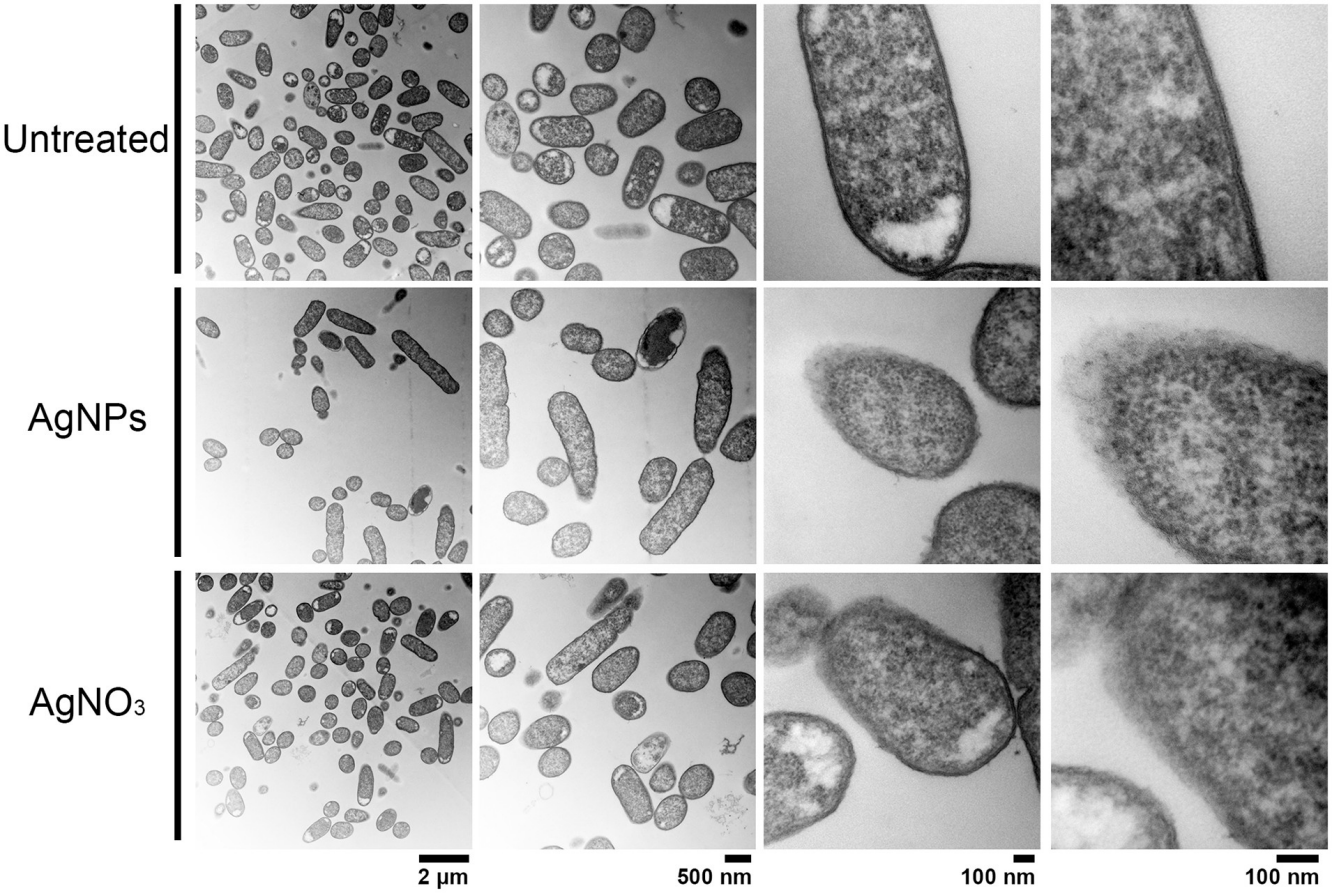
Structural analysis by Transmission Electron Microscopy (TEM) of *S*. Typhimurium exposed to AgNPs or AgNO_3_ (6 and 4 μg.mL^-1^, respectively). Untreated cells were used as control. Representative images of 3 biological replicates are shown.

**Fig 4:**
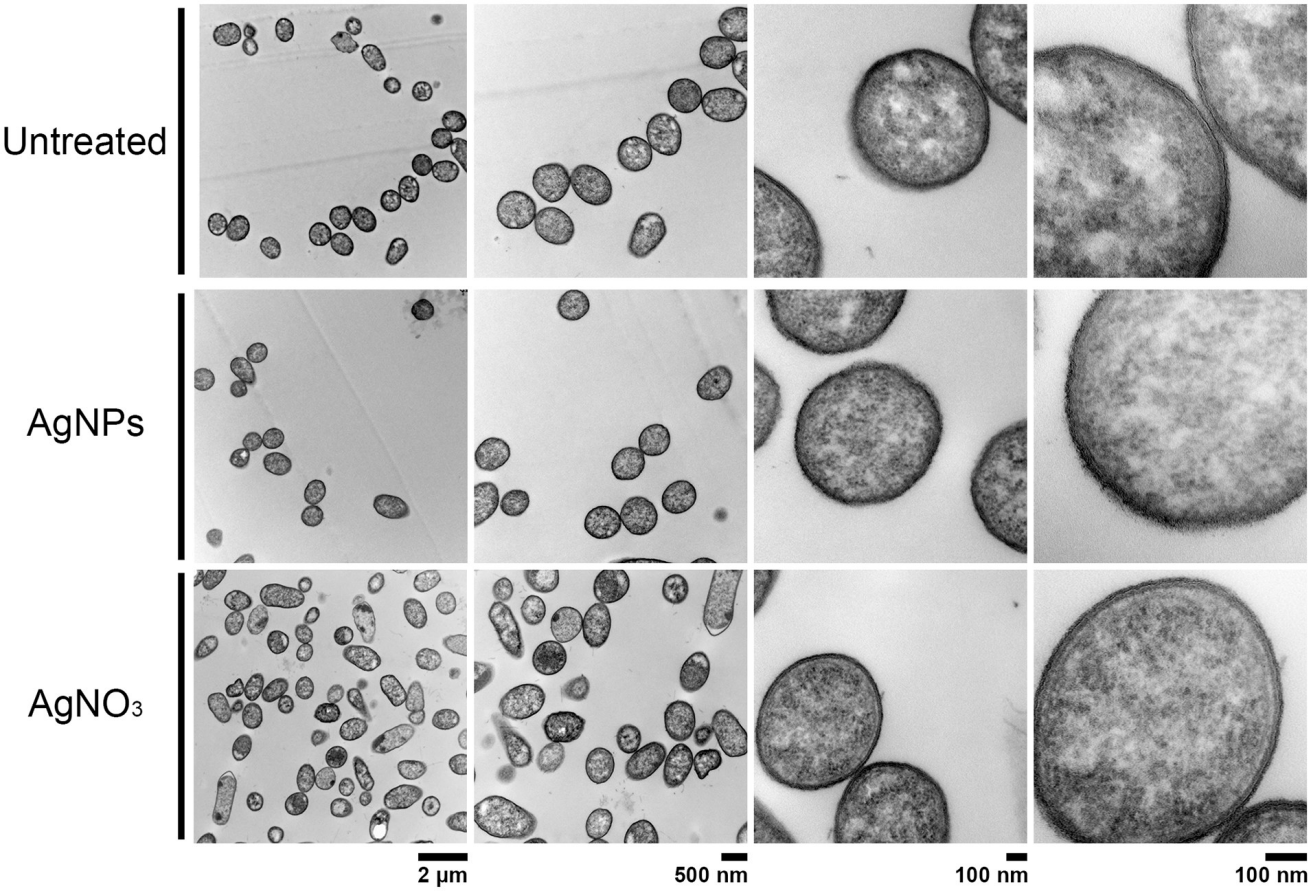
Structural analysis by Transmission Electron Microscopy (TEM) of *S*. *aureus* cells exposed to AgNPs or AgNO_3_ (6 and 4 μg.mL^-1^, respectively). Untreated cells were used as control. Representative images of 3 biological replicates are shown.

**Fig 5:**
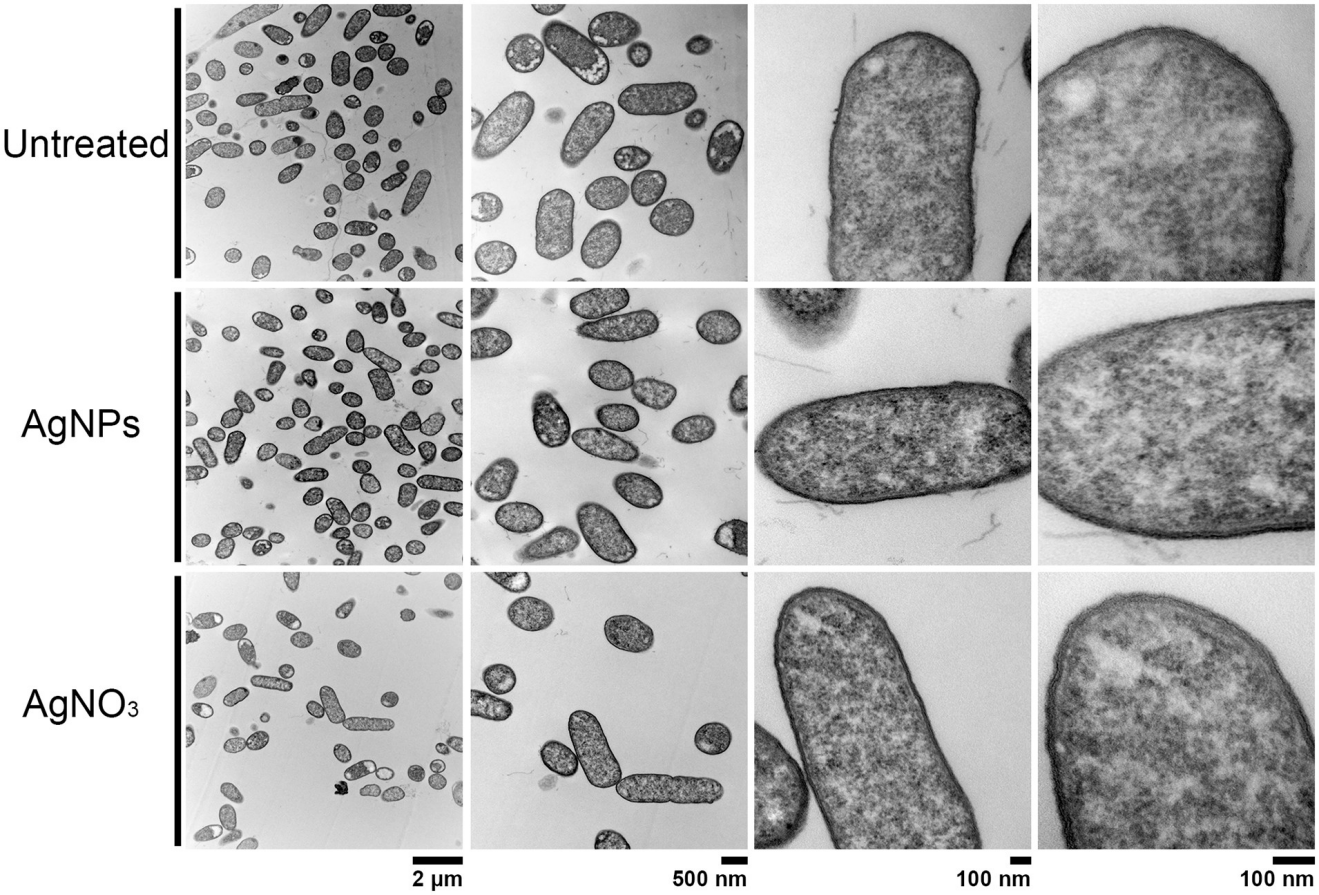
Structural analysis by Transmission Electron Microscopy (TEM) of *B*. *subtilis* exposed to AgNPs or AgNO_3_ (6 and 4 μg.mL^-1^, respectively). Untreated cells were used as control. Representative images of 3 biological replicates are shown.

In order to compare the effect on cell membrane integrity of the NPs, bacterial strains were treated with sub-lethal AgNPs and AgNO_3_ concentrations, and cellular morphology was visualized with TEM (Figs 2–5). In comparison with untreated cells, both AgNPs and AgNO_3_ treatments disrupt the *E*. *coli* and *S*. Typhimurium cell membrane, as can be seen by a loss of integrity of the cell membrane suggesting a direct effect of silver ions on the cell membrane stability (Figs 2 and 3). Interestingly, for *B*. *subtilis* and *S*. *aureus* (both Gram–positive), exposure to sub-lethal concentrations of AgNPs or AgNO_3_, did not cause apparent damage (Figs 4 and 5).

For *E*. *coli*, *S*. Typhimurium and *S*. *aureus*, sublethal concentrations of the combined treatments AgNPs-Km and AgNPs-Cm showed a synergistic and an additive effect, respectively (Table 2, Fig 1). As expected, TEM analysis under these two combined treatments showed dramatic cellular damage validating MIC assays and indicating bacterial cell death (S5 Fig).

### Effect of AgNPs on cell membrane potential and permeability

**Fig 6:**
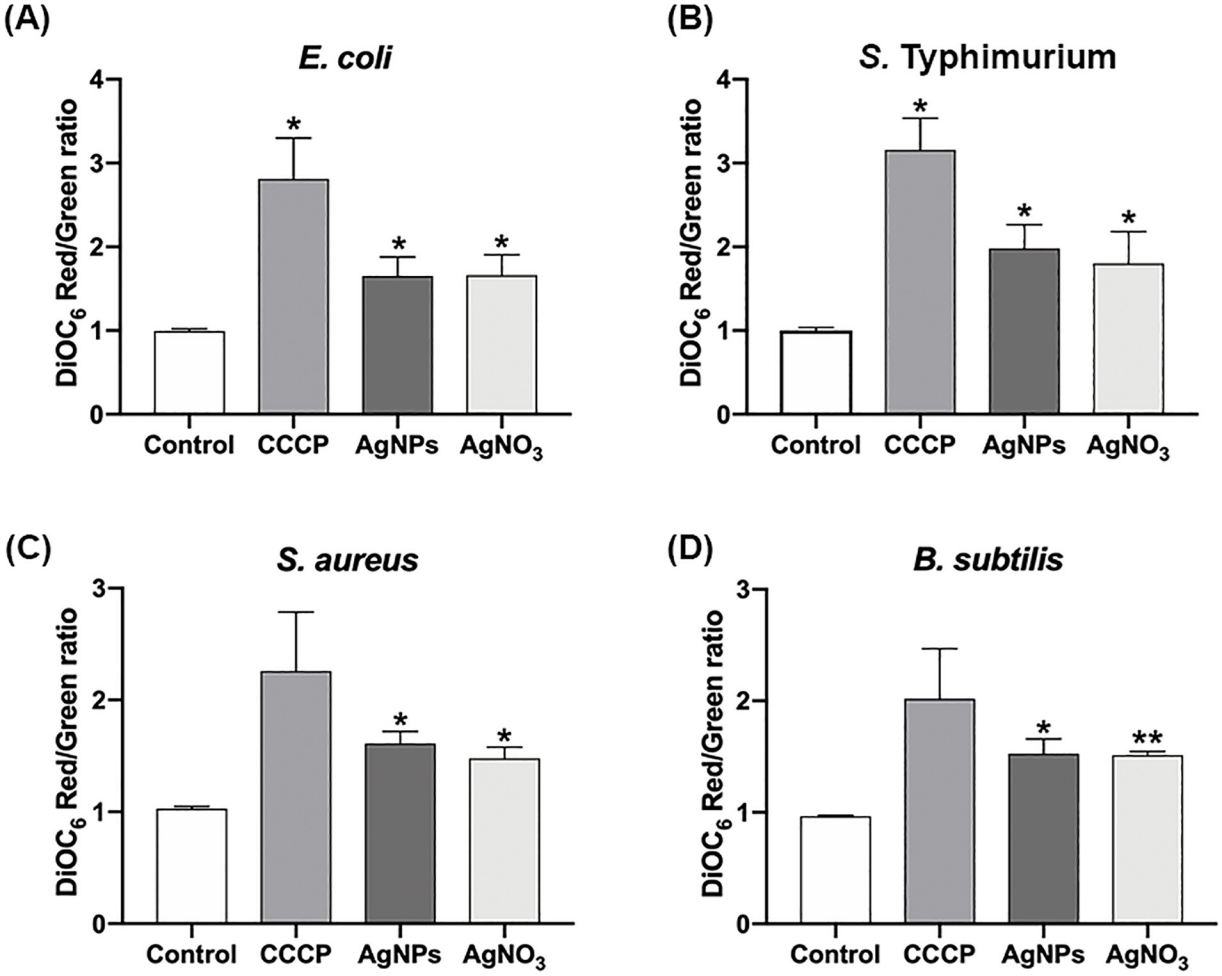
Effect of AgNPs and AgNO_3_ on cell membrane polarity of (A) *E*. *coli*, (B) *S*. Typhimurium, (C) *S*. *aureus*, and (D) *B*. *subtilis*. CCCP was used as depolarized agent control. Treatments were performed by triplicates. Significant changes are marked with an asterisk: * p-value <0.05, ** p-value <0.01 using a 1-way ANOVA with Dunnett´s post test.

**Fig 7:**
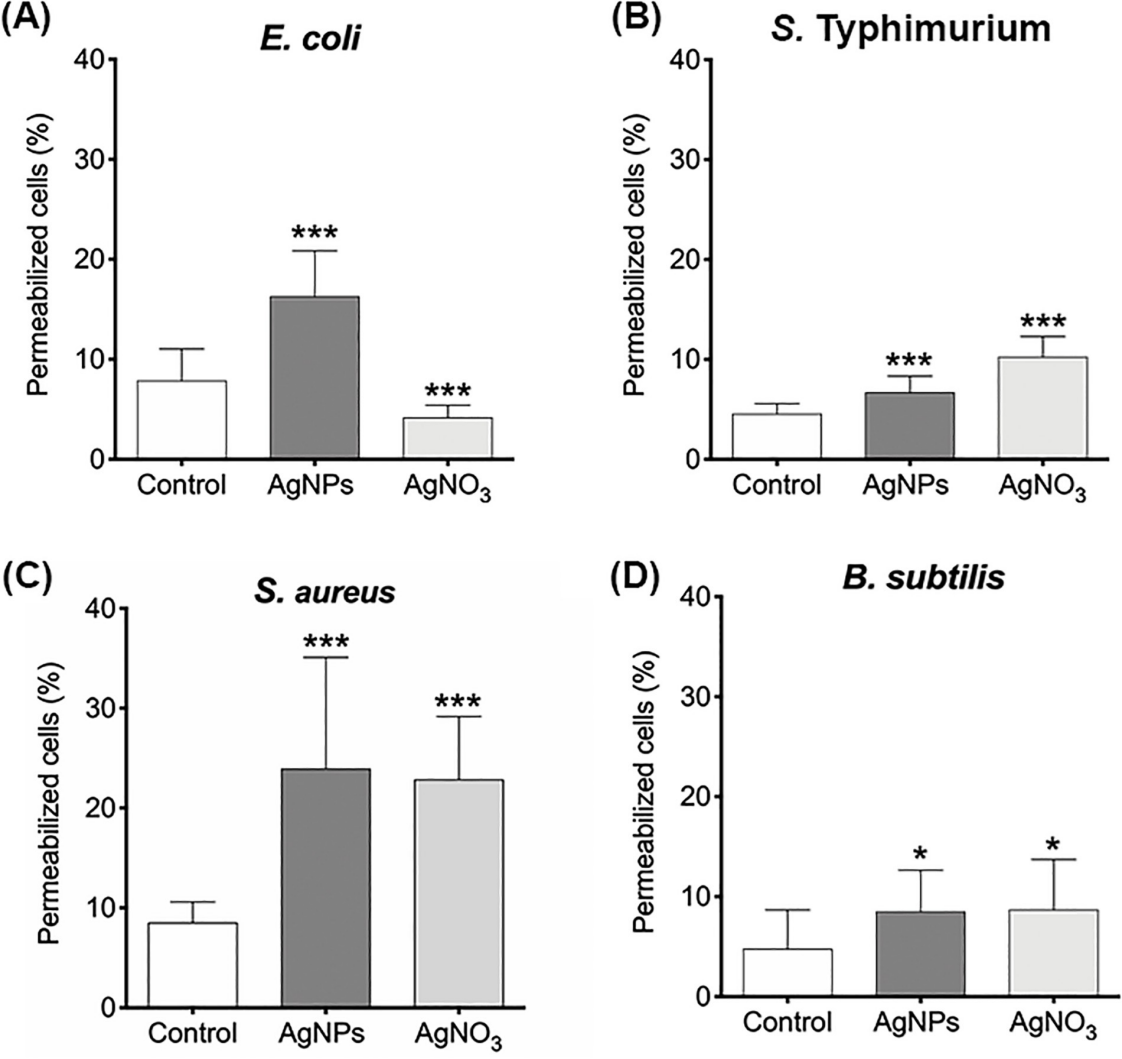
AgNPs and AgNO_3_ permeabilize cell membrane. (A) *E*. *coli*, (B) *S*. Typhimurium, (C) *S*. *aureus*, and (D) *B*. *subtilis*. Propidium iodide intake was quantified as permeabilization marker. Treatments were performed in triplicates. Significant changes are marked with an asterisk: * p-value <0.05, *** p-value <0.01 using a 1-way ANOVA with Dunnett´s posttest.

Gram-negative membrane damage could explain the bactericidal effect of AgNPs when being combined with Km or Cm. However, for *S*. *aureus* no structural modification were observed but a significant synergistic and additive effect was detected for Km and Cm, respectively. Previous reports suggested that AgNPs and AgNO_3_ cause membrane depolarization and destabilization leading to the microbicidal effect of AgNPs and AgNO_3_ [45]. To evaluate membrane depolarization and permeability, we analyzed bacterial cells treated with sub-lethal concentration of AgNPs or AgNO_3_ by flow cytometry. Interestingly, AgNPs and AgNO_3_ depolarized bacterial membrane after 30 min of exposure, and these effects are indistinctly of Gram-negative and Gram-positive bacteria (Fig 6). Moreover, permeability was also altered by changes in membrane potential in cells exposed to AgNPs and AgNO_3_ in all strains tested (Fig 7) [47].

## Discussion

In agreement with previous comparative analysis, the minimal inhibitory concentrations (MIC) of AgNPs and AgNO_3_ were similar in all tested bacterial strains, regardless of their physiological or structural differences, with most MIC values between 6–12 μg.mL^-1^, and the same NPs concentration were determined for the minimal bactericidal concentrations (MBC) (Table 1) [42]. On the contrary, antibiotic susceptibility were variable between strains.

TEM analysis showed that AgNPs significantly affect the cell membrane integrity in Gram-negative bacteria such as *E*. *coli* and *S*. Typhimurium (Figs 2 and 3). However, in treatments on Gram-positive bacteria (*S*. *aureus* and *B*. *subtilis*), no cell wall disruption was observed (Figs 4 and 5). On the other hand, sub-lethal AgNPs concentrations depolarized cell membrane in all bacteria analyzed, which leads to an increase in cell permeability (Fig 6), regardless of the difference in cell wall composition between Gram-positive and Gram-negative bacteria [23,25,36,48,49]. These increases in membrane permeability might facilitate antibiotics cellular entrance, allowing an intracellular antibiotics higher efficiency (Fig 7).

AgNPs and antibiotics combined treatments have been studied previously but no mechanism of action has been established since different phenotypes have been detected between studies [22,33,37]. Moreover, the effect of AgNPs-antibiotics activity can vary in different organisms [50]. These discrepancies found on synergy AgNPs-antibiotics can be explained by different factors: the stabilizing agent used and its relative concentration, which can influence the ability of interaction between AgNPs and antibiotics [34,49,51]. AgNPs functionalized with PVP showed better synergistic antibacterial capacity with antibiotics than those stabilized with citrate or SDS [51]. Moreover, interaction of citrate-stabilized AgNPs and ampicillin has been reported [34], while AgNPs stabilized with PVP did not show such interaction (this work), which suggests that stabilizer plays a central role in this type of interactions, and concomitantly on the antimicrobial interaction (synergistic, additive or no effect).

In this study, AgNPs-Km combination showed a synergistic effect on bacterial growth in *E*. *coli*, *S*. Typhimurium and *S*. *aureus*; whereas the AgNPs-Cm combination presented an additive (but still significant) effect on the same bacterial strains (Fig 1 and Table 2). Panacek, *et al* (2015) reported synergistic interaction between antibiotics with different modes of action and AgNPs, including AgNPs-β-lactam antibiotics [49]. However, it is important to note that the *E*. *coli* strain used in this study showed resistance to ampicillin even without AgNPs treatment, and this ampicillin resistance is overcome when combining AgNPs-ampicillin. A similar effect was found for a clinical isolated *S*. *aureus* strain, which showed resistance to Km, however, when combining AgNPs-Km treatment we also observed a synergic effect (Fig 1 and Table 2). The synergy between AgNPs and antibiotics has been also attributed previously to chemical binding between the sulphur group of antibiotics with AgNPs [36,38], but not conclusive experimental evidence has been provided to support this hypothesis. Deng *et al*. reported a synergistic effect and interaction between AgNPs and several antibiotics (enoxacin, kanamycin, neomycin, and tetracycline). They proposed that this interaction promote an increase in the Ag ions release, which concomitantly increases bacterial growth inhibition in *Salmonella sp*. [34]. However, our results showed no changes in the FT-IR experiments indicating no covalent chemical interaction between AgNPs and the antibiotics tested (S2 Fig). For DLS analysis, superficial charge modification was detected on AgNPs in combination with Km or Amp, suggesting electrostatic interaction. Additionally, AgNPs-Km combination also showed an increase in the hydrodynamic ratio size (S3 Fig). In TEM images, AgNPs-Km or AgNPs-Amp agglomeration was observed (S4 Fig). Nevertheless, no direct correlation between the enhanced antimicrobial effect and the changes in AgNPs interaction, size or charge was found. Thus, it seems that these characteristics are not essential for the bactericidal effect of combined treatments. Interestingly, consistently with other studies, AgNPs alter membrane integrity and increased cell permeability (Figs 6 and 7) [15,24,25].

**Fig 8:**
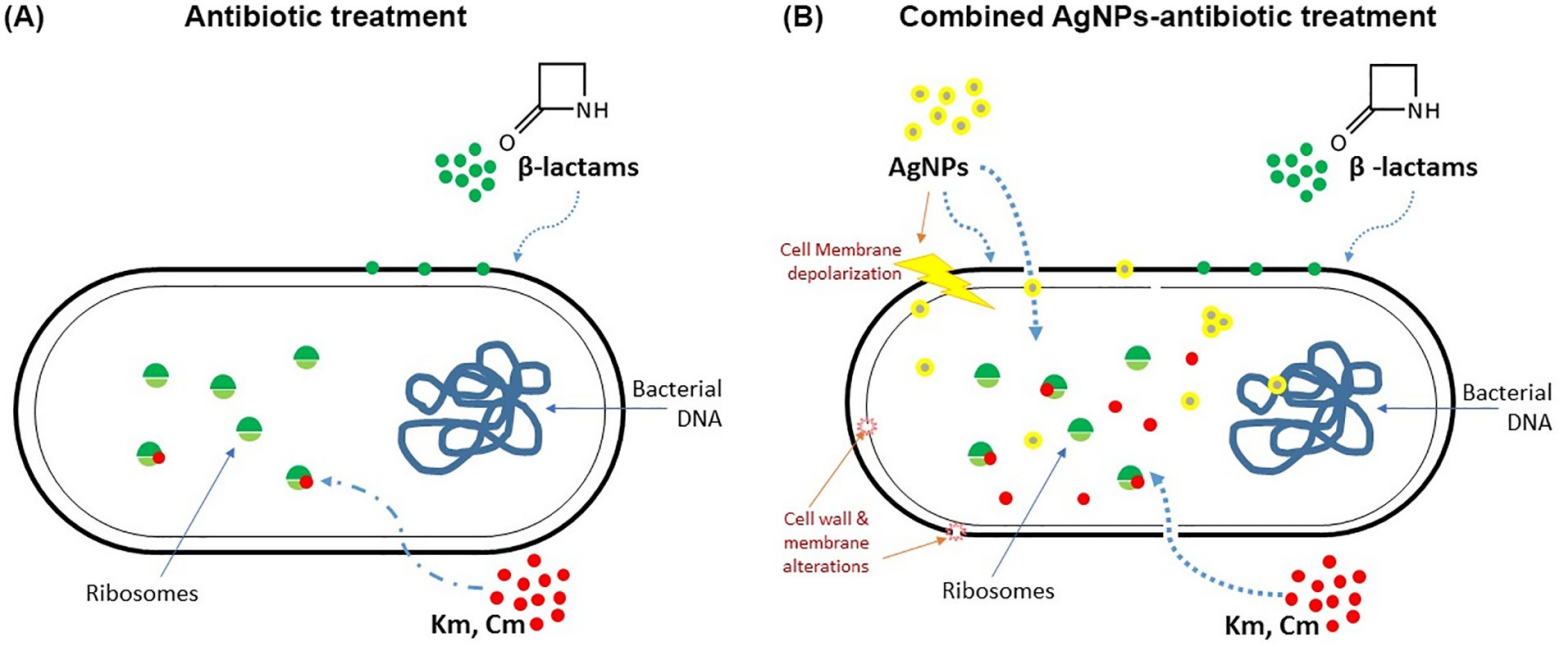
Model of the mechanism of action of combined AgNPs-antibiotic treatment. A) β-lactam antibiotics activity, as well as kanamycin (Km) and chloramphenicol (Cm) are shown. B) In combined treatments, AgNPs depolarize cell membrane affecting permeability and allowing Km and Cm to reach ribosomes inside the cell and increasing their antibacterial activity (synergistic and additive effect).

Here, we propose a mechanism of action of combined AgNPs-antibiotic treatments, where AgNPs destabilize bacterial cell membrane, promote antibiotic internalization in the cell and concomitantly the microbicidal activity (Fig 8). For those antibiotics that act inside the cell, a synergistic or additive effect can be observed because AgNPs facilitate antibiotic cell entrance and promote the access to their target (for Km and Cm, the ribosome) [52]. In contrast, β-lactam antibiotics showed no synergy with AgNPs since these antibiotics act by affecting the integrity of the bacterial cell wall, so their effect is not influenced by changes in cell permeability and the antibiotic cell entrance.

Our model is also supported by Deng´s findings, where four intracellular antibiotics showed synergy in combination with AgNPs [34]. Also, a recent report describes an enhanced activity of ciprofloxacin and gentamicin, among others, with both synergistic and additive effects, when applied in combination with AgNPs [48]. It is important to emphasize that the enhancement effect was higher in antibiotic-resistant strains than in antibiotic-susceptible strains, which can be explained by the model proposed in our study where AgNPs affect the cell membrane and cell wall integrity favoring antibiotic action, leading to a “restored” susceptibility for some antibiotic resistant strains [48].

A physiological mechanism of the synergistic effect of AgNPs and antibiotic combinations is herein explained with experimental support. Our results help to elucidate how the synergistic effect of these combinations occurs. According to our experimental data, the enhancement of antibiotics antimicrobial activity is due to the effect of AgNPs on the cell structure rather than on the AgNPs-antibiotic direct interaction.

Understanding the mechanism of action of antibiotics, AgNPs and combined treatments allows predicting a more feasible and improving treatments or, the design of new ones more efficient. Even if some aspects of the mechanism of action remains unknown, our results contribute to provide a more effective way to fight infectious diseases. The enhancement of the antimicrobial activity due to AgNPs-antibiotic combinations would enable the use of antibiotics that have fallen into disuse because of bacterial resistance issues, providing additional treatment possibilities in health care, veterinary and agriculture sectors. Thus, nanoantibiotics have a potential impact on social and economic problems, as they may help mitigate the current crisis due to antibiotic resistance.

## Supporting information

S1 Fig Silver nanoparticles characterization. A) UV-Vis profile. B) TEM micrograph of AgNPs. C) Average diameter of AgNPs. D) Zeta potential analysis.
(PDF)

S2 Fig FT-IR analysis of the AgNPs, antibiotics and combined treatments. A) Ampicillin (Amp); B) Aztreonam (Azm); C) Kanamycin (Km); and D) Chloramphenicol (Cm). Transmission spectra of the AgNPs-antibiotic combinations were similar to those of non-combined AgNPs and antibiotics.
(PDF)

S3 Fig DLS analysis of the combined AgNPs and antibiotic treatments at 0 (red) and after 24 hours of incubation (green). The charge (A) and size (B) of AgNPs are different for each antibiotic.
(PDF)

S4 Fig TEM images of the combined AgNPs and antibiotic treatments. An aggregative effect was observed for Km + AgNPs and Amp + AgNPs combinations (marked with arrows). Images were taken after 24 hours of incubation.
(PDF)

S5 Fig TEM images of bacterial cells exposed to sublethal concentrations of AgNPs and antibiotics. *E*. *coli*, *S*. Typhimurium, *S*. *aureus* and *B*. *subtilis* were exposed to combined treatments for 24 hr. Representative images are shown.
(PDF)

S1 Table Antibiotic sub-letal concentrations (μg.mL^-1^). (PDF)
