# Supplementary material for: Enhancement of antibiotics antimicrobial activity due to the silver nanoparticles impact on the cell membrane
Source: PLoS One. 2019 Nov 8;14(11):e0224904. doi: 10.1371/journal.pone.0224904 (PMC6839893; doi:10.1371/journal.pone.0224904)
Supplement: S1 Fig — A) UV-Vis profile. B) TEM micrograph of AgNPs. C) Average diameter of AgNPs. D) Zeta potential analysis. (PDF) [file pone.0224904.s001.pdf]

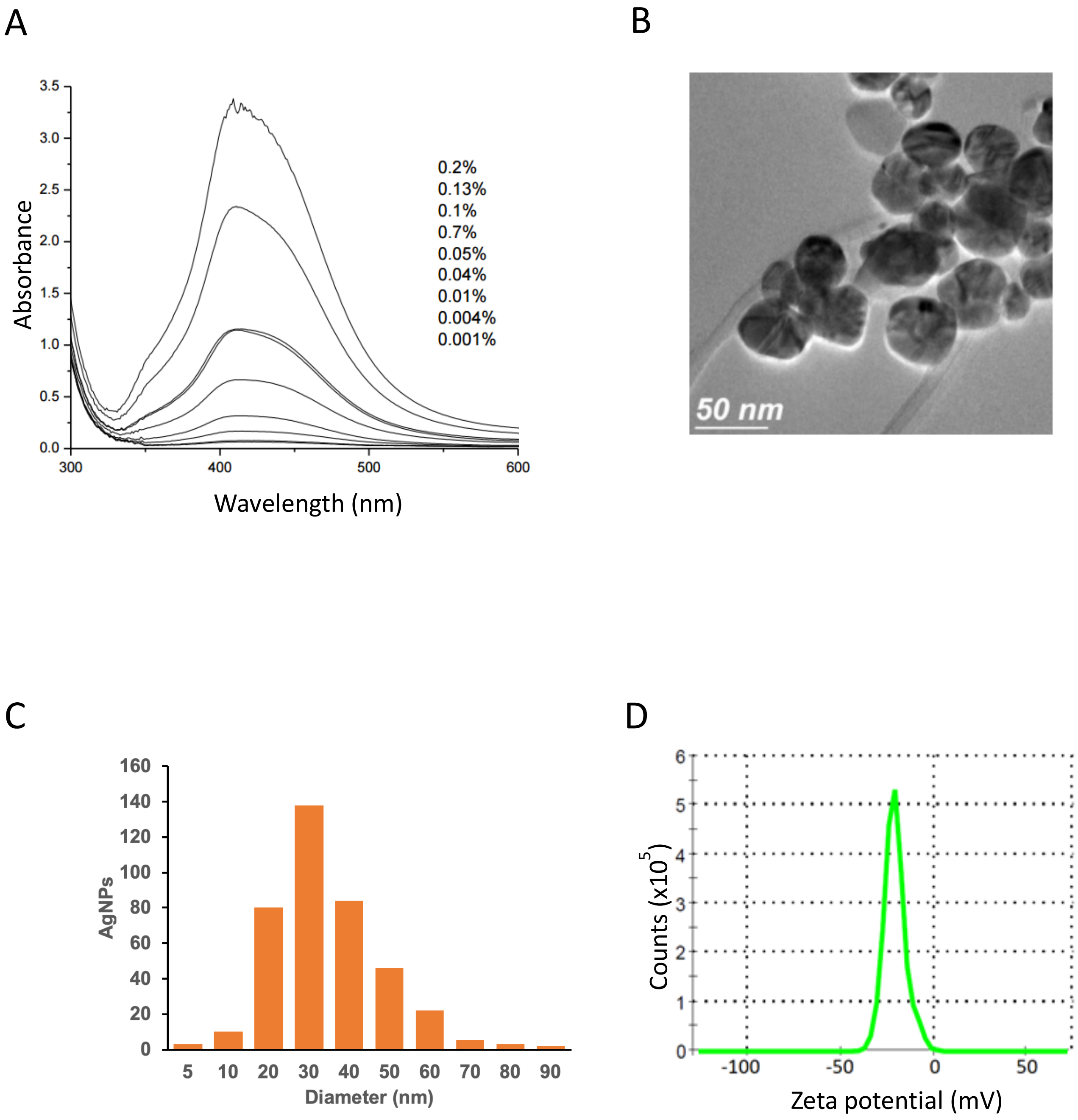

S1 Fig. Silver nanoparticles characterization. A) UV-Vis profile. B) TEM micrograph of AgNPs. C) Average diameter of AgNPs. D) Zeta potential analysis
