# Supplementary material for: Enhancement of antibiotics antimicrobial activity due to the silver nanoparticles impact on the cell membrane
Source: PLoS One. 2019 Nov 8;14(11):e0224904. doi: 10.1371/journal.pone.0224904 (PMC6839893; doi:10.1371/journal.pone.0224904)
Supplement: S2 Fig — A) Ampicillin (Amp); B) Aztreonam (Azm); C) Kanamycin (Km); and D) Chloramphenicol (Cm). Transmission spectra of the AgNPs-antibiotic combinations were similar to those of non-combined AgNPs and antibiotics. (PDF) [file pone.0224904.s002.pdf]

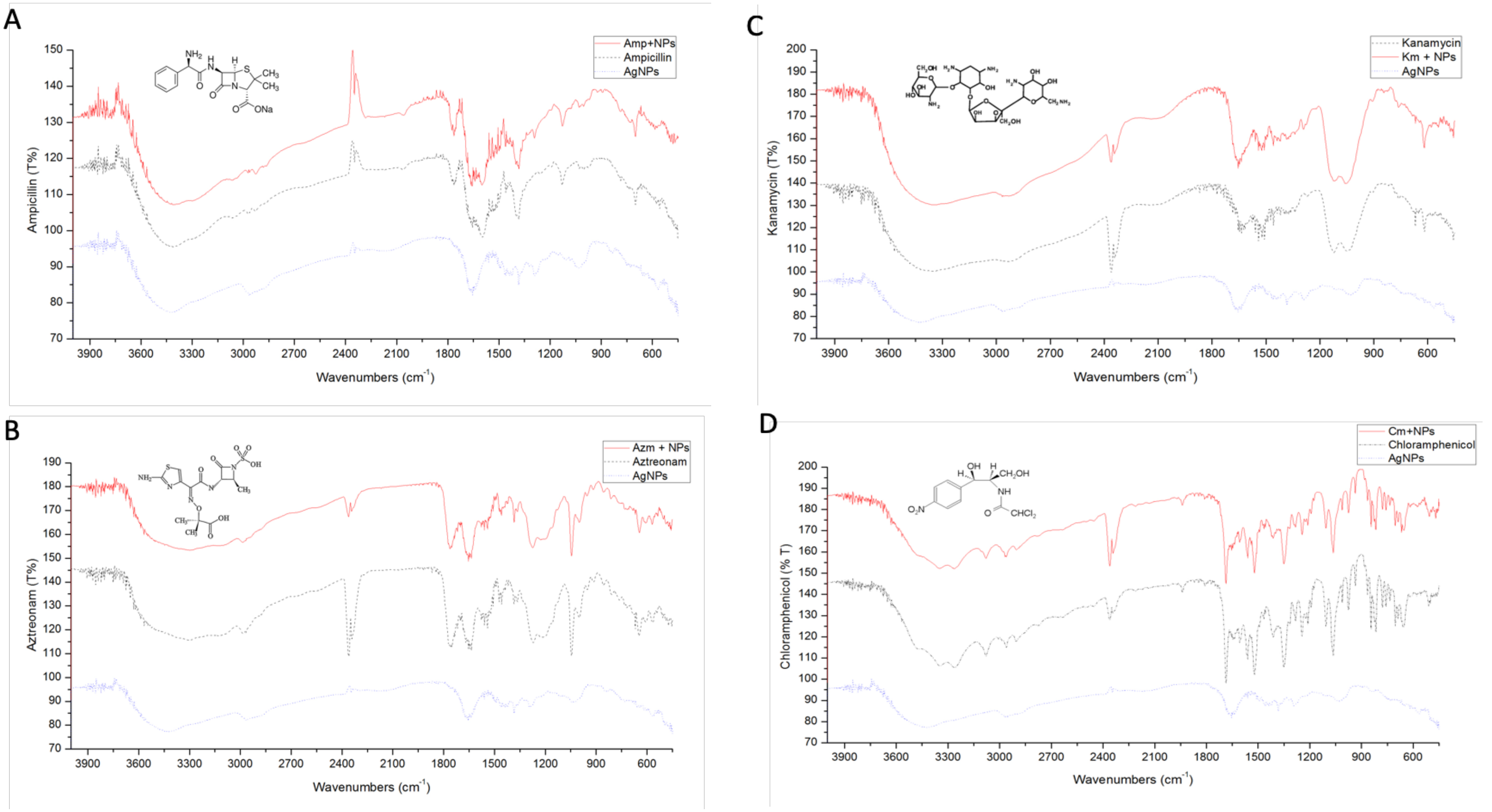

S2 Fig. FT-IR analysis of the AgNPs, antibiotics and combined treatments. A) Ampicillin (Amp); B) Aztreonam (Azm); C) Kanamycin (Km); and D) Chloramphenicol (Cm). Transmission spectra of the AgNPs-antibiotic combination were similar to those of the non-combined AgNPs and antibiotics.
