# Supplementary material for: Enhancement of antibiotics antimicrobial activity due to the silver nanoparticles impact on the cell membrane
Source: PLoS One. 2019 Nov 8;14(11):e0224904. doi: 10.1371/journal.pone.0224904 (PMC6839893; doi:10.1371/journal.pone.0224904)
Supplement: S3 Fig — The charge (A) and size (B) of AgNPs are different for each antibiotic. (PDF) [file pone.0224904.s003.pdf]

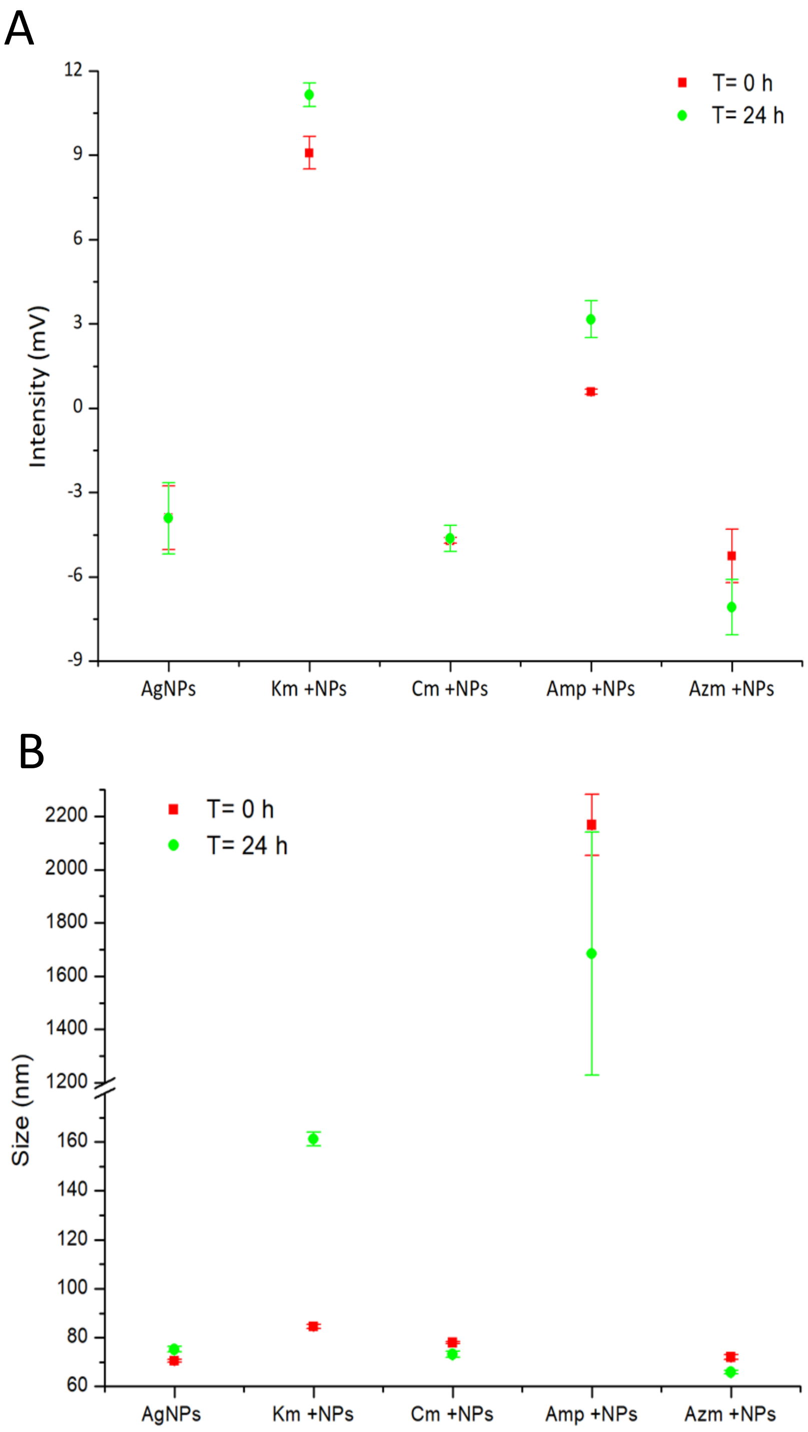

S3 Fig. DLS analysis of the combined AgNPs and antibiotic treatments at 0 (red) and after 24 hours of incubation (green). The charge (A) and size (B) of AgNPs are different for each antibiotic.
