# Supplementary material for: Enhancement of antibiotics antimicrobial activity due to the silver nanoparticles impact on the cell membrane
Source: PLoS One. 2019 Nov 8;14(11):e0224904. doi: 10.1371/journal.pone.0224904 (PMC6839893; doi:10.1371/journal.pone.0224904)
Supplement: S4 Fig — An aggregative effect was observed for Km + AgNPs and Amp + AgNPs combinations (marked with arrows). Images were taken after 24 hours of incubation. (PDF) [file pone.0224904.s004.pdf]

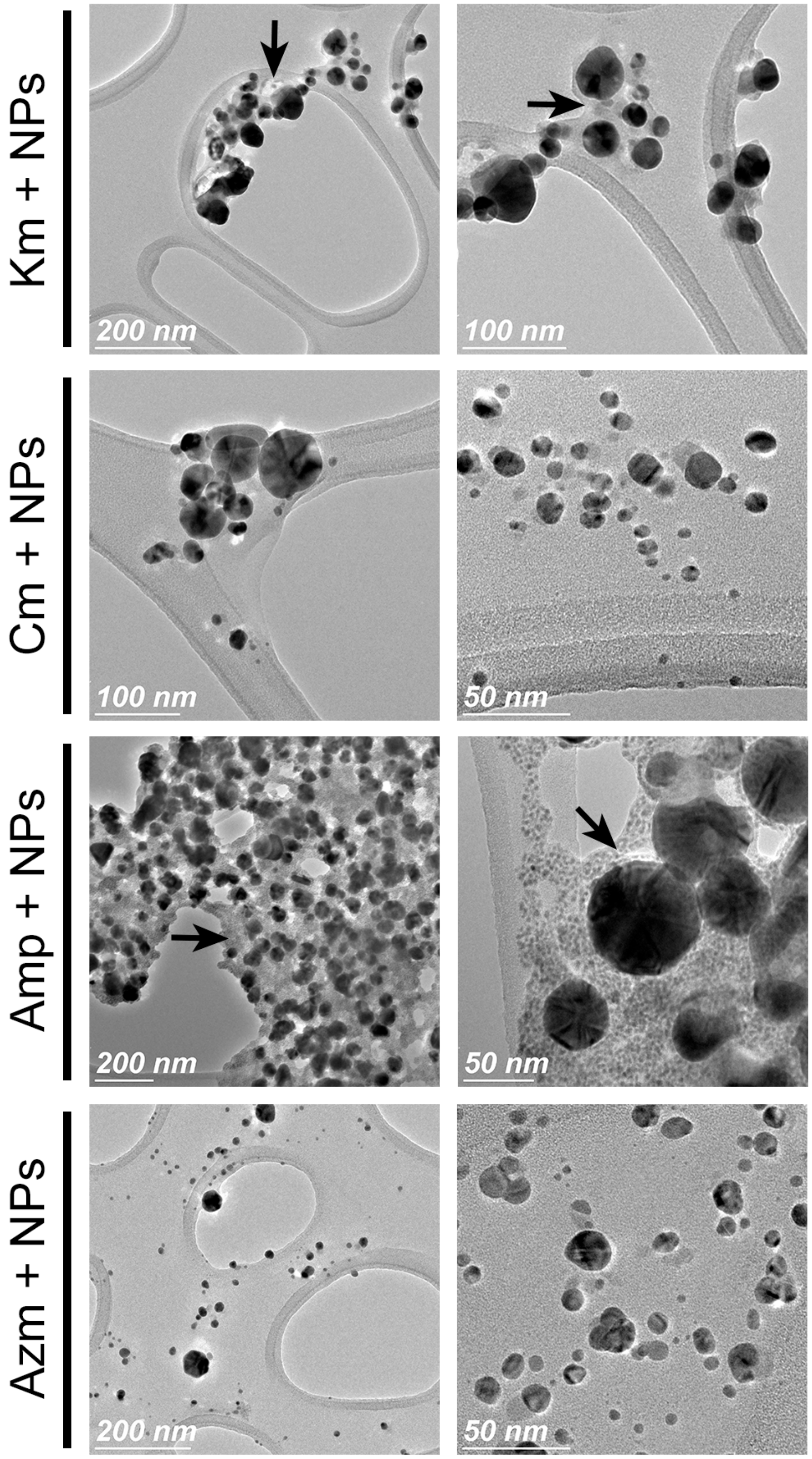

S4 Figure. TEM images of the combined AgNPs and antibiotic treatments. An aggregative effect was observed for Km + AgNPs and Amp + AgNPs combinations (marked with arrows). Images were taken after 24 hours of incubation.
