# Supplementary material for: Enhancement of antibiotics antimicrobial activity due to the silver nanoparticles impact on the cell membrane
Source: PLoS One. 2019 Nov 8;14(11):e0224904. doi: 10.1371/journal.pone.0224904 (PMC6839893; doi:10.1371/journal.pone.0224904)
Supplement: S5 Fig — E. coli, S. Typhimurium, S. aureus and B. subtilis were exposed to combined treatments for 24 hr. Representative images are shown. (PDF) [file pone.0224904.s005.pdf]

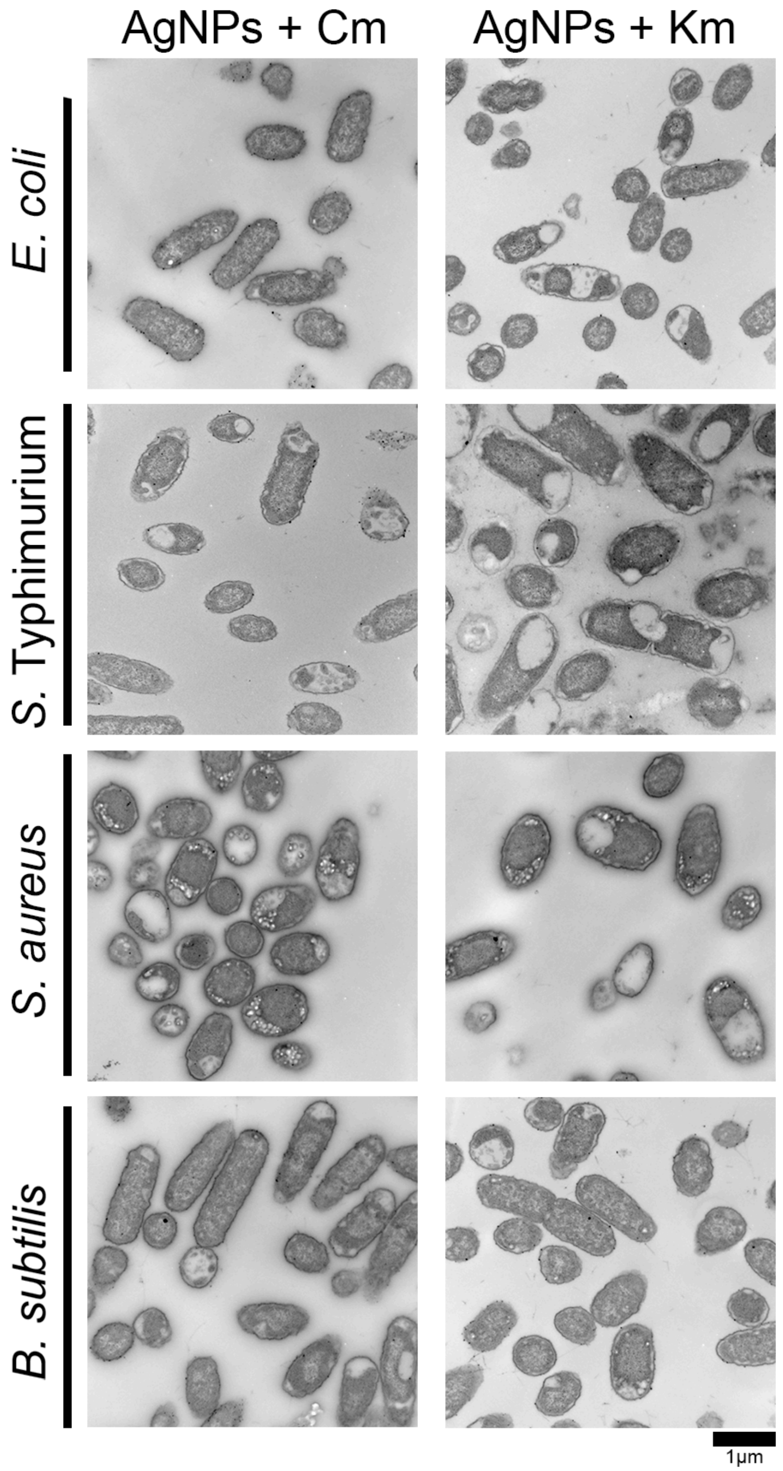


**S5 Fig. TEM images of bacterial cells exposed to sublethal concentrations of AgNPs and antibiotics**. *E. coli*, *S.* Typhimurium, *S. aureus* and *B. subtilis* were exposed to combined treatments for 24 hr. Representative images are shown.
