# Supplementary material for: Enhancement of antibiotics antimicrobial activity due to the silver nanoparticles impact on the cell membrane
Source: PLoS One. 2019 Nov 8;14(11):e0224904. doi: 10.1371/journal.pone.0224904 (PMC6839893; doi:10.1371/journal.pone.0224904)
Supplement: S1 Table — (PDF) [file pone.0224904.s006.pdf]

Strain	**Cm**	**Km**	**Amp**	**Bpm**	**Azm**
E. coli	0.35	0.05	1	0.02	0.05
***S.* Typhimurium**	0.5	0.1	0.25	0.02	0.02
S. aureus	0.05	16	0.25	0.02	0.02
B. subtilis	8	0.12	0.05	0.005	16

Cm= chloramphenicol; Km= kanamycin; Amp= ampicillin; Bpm= biapenem, Azm= aztreonam.

S1 Table. Antibiotic sub-letal concentrations (µg.ml^{-1}).
